# Seven Synchronous Primary Melanomas on the Back

**DOI:** 10.5826/dpc.1101a104

**Published:** 2020-01-29

**Authors:** Sanja Javor, Simona Sola, Alexandra M.G. Brunasso, William Bruno, Cesare Massone

**Affiliations:** 1 Dermatology Unit, Galliera Hospital, Genoa, Italy; 2 Surgical Pathology, Galliera Hospital, Genoa, Italy; 3 Genetics of Rare Cancers, Department of Internal Medicine and Medical Specialties (DiMI), University of Genoa and IRCCS Ospedale Policlinico San Martino, Genoa, Italy

**Keywords:** synchronous melanomas, chronic sun exposure, BRAF, dermoscopy

## Introduction

Up to 5% of patients may get multiple cutaneous primary melanomas. The clinical diagnosis of synchronous melanoma (SM) is rare, and it appears to be more frequent in persons older than 50 years [1]. Multiple cutaneous primary melanomas that are diagnosed within 3 months of the first malignancy are considered synchronous, whereas a development of a second primary melanoma during a longer follow-up period is considered metachronous.

## Case Presentation

**Figure 1:**
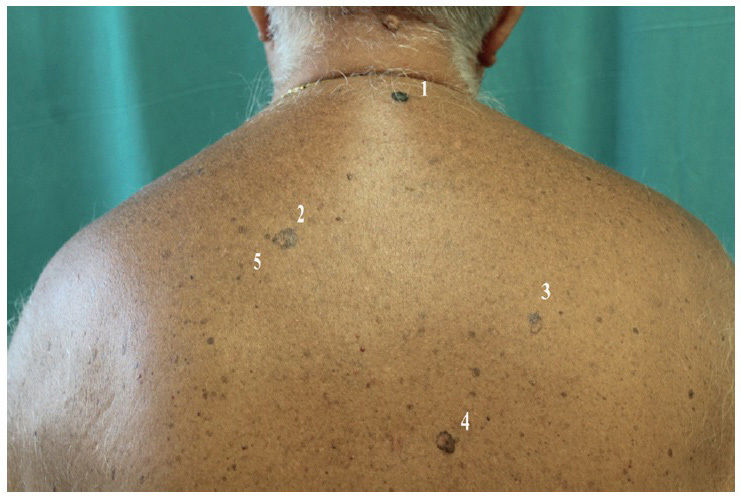
Severe chronically sun-damaged skin on the back of a 74-year-old man with several pigmented atypical lesions. Lesion #1 was a nodular melanoma with a Breslow thickness of 4 mm. Lesions #2–5 were melanomas in situ.

**Figure 2:**
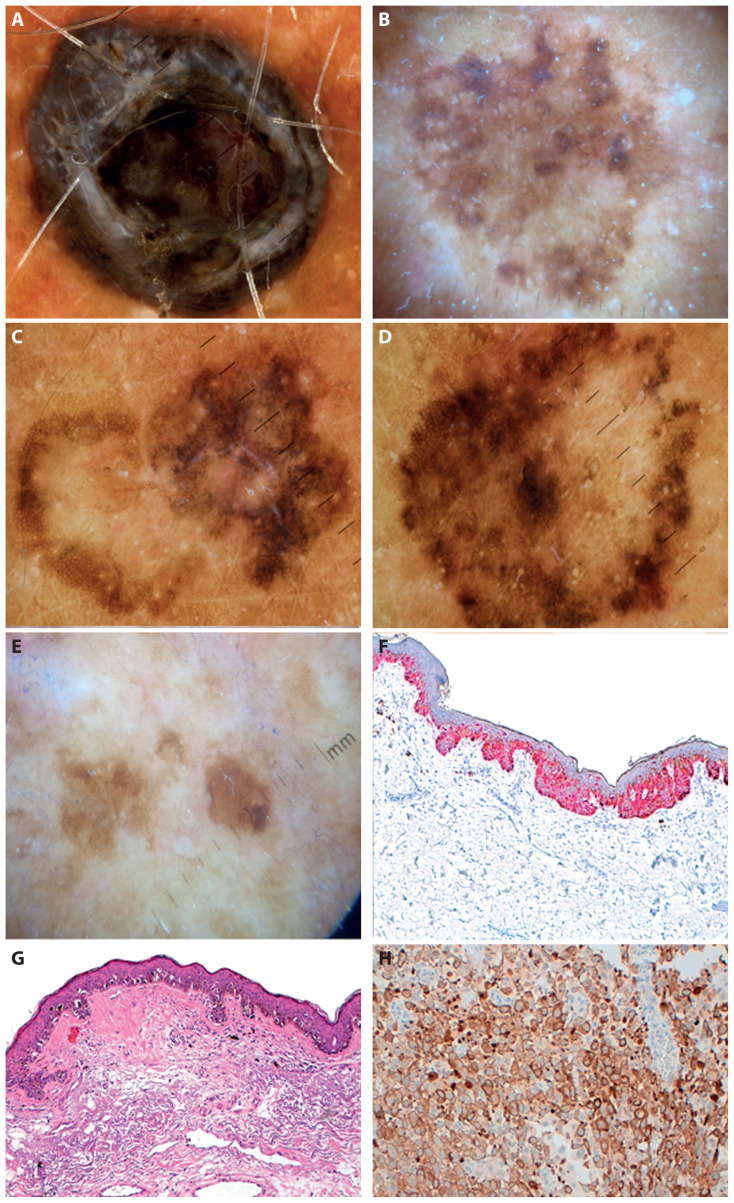
(A) Polarized dermoscopy of lesion #1 (Figure 1) showed a structureless pattern with black-blue color and shiny white structures. (B) Polarized dermoscopy of lesion #2 (Figure 1). (C) Polarized dermoscopy of lesion #3 (Figure 1). (D) Polarized dermoscopy of lesion #4 (Figure 1). (E) Polarized dermoscopy of lesion #5 (Figure 1). Dermoscopy shows regression (scar-like areas) (B–E), atypical network (B–E), asymmetry in structure and/or in color in 1 or 2 axes of the lesion (B–E), and irregular dots (C–D). (F) Melan-A staining of the melanoma in situ (sixth melanoma) incidental finding of the wide excision of lesion #1 (Figure 1); incidental finding of the wide excision of lesion #1. Melan-A shows atypical melanocytes in all epidermal layers. There is no continuity with the scar of the previous excision (original magnification ×100). (G) Melanoma in situ (seventh melanoma), incidental finding of the wide excision of lesion #2 (Figure 1); atypical melanocytes in single units along the dermoepidermal junction and upper layers of the epidermis; and in the dermis melanophages and solar elastosis. There is no continuity with the scar of the previous excision (H&E; original magnification ×100). (H) Positivity for p53 staining in the nodular melanoma in lesion #1 (Figure 1); original magnification ×400.

A 74-year-old Caucasian man with Fitzpatrick III skin type complained of a bleeding nodule on his nuchal region (Figure 1, lesion #1) that had been growing progressively for 12 months. He denied any personal or family history of melanoma. He reported chronic sun exposure from a young age, with episodes of sunburns in childhood. His occupational status was retired, and he denied working outdoors. Past medical history revealed a papillary thyroid microcarcinoma 10 years prior. Medications included only losartan and levothyroxine. Skin examination revealed a black-bluish nodule of 10 × 9 mm in his nuchal region with signs of ulceration. Upon polarized dermoscopy, a structureless pattern with black-blue color and shiny white structures was observed (Figure 2A). Four other suspected melanocytic lesions were observed on his back (Figure 1, lesions #2–5). On dermoscopy they showed regression (scar-like areas) (Figure 2, B–E), atypical network (Figure 2, B–E), asymmetry in structure and in color in 1 or 2 axes of the lesion (Figure 2, B–E), irregular pigmented areas (Figure 2, B–D) [2], and irregular dots (Figure 2, C–D). The histopathological examination of lesion #1 showed a dense nodular infiltration of sheets of atypical melanocytes with atypical mitosis (5/mm^2^) and ulceration in the papillary and reticular dermis. Adjacent to the nodular tumor, an epidermal collarette was visible without adjacent intraepidermal or micro-invasive radial growth phase. Melanocytes stained positive for S100, Melan-A, HMB-45 and p53 (Figure 2H). Lesion #1 was diagnosed a T4b nodular melanoma with a Breslow thickness of 4 mm. Lesions #2–5 showed atypical melanocytes and irregular nests in all epidermal layers with melanophages and solar elastosis in the dermis and were diagnosed as melanomas in situ. No association with nevus was documented, but solar elastosis was reported in all melanomas.

Sentinel lymph node biopsy of the left axillary region together with wide excisions were performed, and the sentinel lymph node biopsy did not reveal any invasion of lymph nodes. The histopathology of the excisions with 2-cm margins of the previously *in sano* excised nodular melanoma (Figure 1, lesion #1) and excision of 5 mm margins of previously *in sano* excised melanoma in situ (Figure 1, lesion #2) revealed 2 in situ melanomas as incidental findings within the same samples (Figure 2, F and G). Both melanomas in situ were not in continuity with the scar and were not detected clinically nor dermoscopically. In all melanomas, real-time PCR did not disclose any *BRAF* mutation; melanoma #1 was *KRAS* and *NRAS* (lesions #2, 3, 4) wild type. Next-generation sequencing did not reveal any pathogenic variant in *BAP1, CDKN2A*, *CDK4*, *MITF*, or *POT1* genes. Staging investigations were negative.

## Conclusions

In our review of the English literature, in applying strict temporal criteria for the definition of SM (within 3 months from first melanoma), we found only 4 reports of patients having >3 SMs (only 1 with 5 SMs) [3]. All the reported melanomas were located on different body regions [3], but in our patient were found only on his back. This fact, together with the synchronous occurrence, absence of genetic mutations, the presence of solar elastosis, overexpression of p53 in the nodular melanoma, the similar dermoscopic findings for the melanomas in situ, and the absence of *BRAF* mutations in all melanomas support a strong pathogenetic role of chronic instead of intermittent sun exposure. Mutations of p53 have been reported in about 25% of thick melanomas and appear late during the evolution of melanoma, suggesting that UV radiation-induced mutations steadily increase to melanoma invasion [4].
